# ^18^F-FDG Pet Parameters and Radiomics Features Analysis in Advanced Nsclc Treated with Immunotherapy as Predictors of Therapy Response and Survival

**DOI:** 10.3390/cancers12051163

**Published:** 2020-05-05

**Authors:** Giulia Polverari, Francesco Ceci, Valentina Bertaglia, Maria Lucia Reale, Osvaldo Rampado, Elena Gallio, Roberto Passera, Virginia Liberini, Paola Scapoli, Vincenzo Arena, Manuela Racca, Andrea Veltri, Silvia Novello, Désirée Deandreis

**Affiliations:** 1 Division of Nuclear Medicine, Department of Medical Sciences, University of Turin, AOU Città della Salute e della Scienza, 10126 Turin, Italy; giuliapolverari@yahoo.com (G.P.); passera.roberto@gmail.com (R.P.); v.liberini@gmail.com (V.L.); desiree.deandreis@unito.it (D.D.); 2 PET/CT Center, Affidea IRMET, 10135 Turin, Italy; vincenzo.arena@affidea.it; 3 Department of Oncology, University of Turin, San Luigi Gonzaga Hospital, Orbassano, 10043 Torino, Italy; valentina.bertaglia@gmail.com (V.B.); realemarialucia@gmail.com (M.L.R.); silvia.novello@unito.it (S.N.); 4 Medical Physics Unit, S.C. Fisica Sanitaria, A.O.U. Città della Salute e della Scienza, 10135 Turin, Italy; orampado@cittadellasalute.to.it (O.R.); gallio.elena@gmail.com (E.G.); 5 Nuclear Medicine, Istituto per la Ricerca e la Cura del Cancro (IRCC), 10060 Candiolo, Italy; paola.scapoli@ircc.it (P.S.); manuela.racca@ircc.it (M.R.); 6 Radiology Unit, Department of Oncology, University of Turin, San Luigi Gonzaga Hospital, Orbassano, 10043 Torino, Italy; andrea.veltri@unito.it

**Keywords:** PET/CT, immunotherapy, PD-1, PD-L1, response to therapy, NSCLC

## Abstract

Objectives: (1.1) to evaluate the association between baseline 18F-FDG PET/CT semi-quantitative parameters of the primary lesion with progression free survival (PFS), overall survival (OS) and response to immunotherapy, in advanced non-small cell lung carcinoma (NSCLC) patients eligible for immunotherapy; (1.2) to evaluate the application of radiomics analysis of the primary lesion to identify features predictive of response to immunotherapy; (1.3) to evaluate if tumor burden assessed by 18F-FDG PET/CT (N and M factors) is associated with PFS and OS. Materials and Methods: we retrospectively analyzed clinical records of advanced NCSLC patients (stage IIIb/c or stage IV) candidate to immunotherapy who performed 18F-FDG PET/CT before treatment to stage the disease. Fifty-seven (57) patients were included in the analysis (F:M 17:40; median age = 69 years old). Notably, 38/57 of patients had adenocarcinoma (AC), 10/57 squamous cell carcinoma (SCC) and 9/57 were not otherwise specified (NOS). Overall, 47.4% patients were stage IVA, 42.1% IVB and 8.8% IIIB. Immunotherapy was performed as front-line therapy in 42/57 patients and as second line therapy after chemotherapy platinum-based in 15/57. The median follow up after starting immunotherapy was 10 months (range: 1.5–68.6). Therapy response was assessed by RECIST 1.1 criteria (CT evaluation every 4 cycles of therapy) in 48/57 patients or when not feasible by clinical and laboratory data (fast disease progression or worsening of patient clinical condition in nine patients). Radiomics analysis was performed by applying regions of interest (ROIs) of the primary tumor delineated manually by two operators and semi-automatically applying a threshold at 40% of SUVmax. Results: (1.1) metabolic tumor volume (MTV) (*p* = 0.028) and total lesion glycolysis (TLG) (*p* = 0.035) were significantly associated with progressive vs. non-progressive disease status. Patients with higher values of MTV and TLG had higher probability of disease progression, compared to those patients presenting with lower values. SUVmax did not show correlation with PD status, PFS and OS. MTV (*p* = 0.027) and TLG (*p* = 0.022) also resulted in being significantly different among PR, SD and PD groups, while SUVmax was confirmed to not be associated with response to therapy (*p* = 0.427). (1.2) We observed the association of several radiomics features with PD status. Namely, patients with high tumor volume, TLG and heterogeneity expressed by “skewness” and “kurtosis” had a higher probability of failing immunotherapy. (1.3) M status at 18F-FDG PET/CT was significantly associated with PFS (*p* = 0.002) and OS (*p* = 0.049). No significant associations were observed for N status. Conclusions: 18F-FDG PET/CT performed before the start of immunotherapy might be an important prognostic tool able to predict the disease progression and response to immunotherapy in patients with advanced NSCLC, since MTV, TLG and radiomics features (volume and heterogeneity) are associated with disease progression.

## 1. Introduction

Lung cancer remains the leading cause of cancer-related death, despite continuous progresses in diagnosis and therapy [1]. Non-small cell lung carcinoma (NSCLC) accounts for 80–85% of all cases of lung cancer [1]. In almost half of the patients, the diagnosis of NSCLC is performed when the disease is already in advanced stages (stage III or stage IV), having an estimated overall 5-year survival rate of 18% [2]. In more than half of patients, the diagnosis of NSCLC is performed when the disease is already in advanced stage with an estimated overall 5-year survival rate of 18% [2,3]. Platinum-based chemotherapy can improve outcome, but the rise of immunotherapy has changed this scenario. Multiple molecules targeting the programmed cell death-1 (PD-1) and the programmed death ligand-1 (PD-L1) axis have been proposed [3]. Different immune checkpoint inhibitors have been approved in the second line setting on the basis of an improving in overall survival (OS) compared to chemotherapy, irrespective to PDL1 expression or with a PD-L1 expression ≥1% for pembrolizumab. Pembrolizumab has also been approved as first-line treatment for advanced NSCLC with PD-L1 higher than 50%, given the significant increase in both progression free survival (PFS) (10.3 versus 6 months) and OS (30 vs. 14.2 months) compared to chemotherapy [3,4]. Moreover, combination strategies of chemo-immunotherapy have recently enriched the upfront therapy approach. However, the benefit with immunotherapy is not seen for the entire population, making the identification of biomarkers and predicting tools a critical step in selecting the ideal candidate population. In NSCLC adenocarcinoma, platinum chemotherapy, plus pemetexered and pembrolizumab, reached a disease control rate of 84.6%, while it was 70.4% for chemotherapy only [5]. However, approximately 15% of positive PD-L1 patients will have disease progression within two months and a median survival of only three months [4]. The inter-patient, inter-tumour and even intra-tumour heterogeneity seem to be the major reasons of such a large prognosis variability, representing a key challenge for the scientific community. In this scenario, the application of new generation image analysis, such as radiomics, might provide further and useful information [6].

Radiomics is an emerging field, defined as the high-throughput extraction of quantitative features from medical images [6,7]. This approach provides high-dimensional data describing the properties of shape and texture of tumors captured in different diagnostic procedures, including PET imaging. The radiomics features are believed to contain information that reflects underlying tumor pathophysiology and allow evaluation of tumor heterogeneity [6,7]. The primary goal of radiomics is to build a clinically relevant predictive, descriptive, or prognostic model using these parameters. Focusing on the relationship between quantitative biological features and cancer prognosis by non-invasive method and aiding clinicians in selecting the appropriate treatments, radiomics is considered a step toward the application of personalized medicine in routine practice. In this respect, NSCLC is a key example to show the advantages of a tailored approach [8,9].

## 2. Materials and Methods

### 2.1. Objectives

The objectives of this study were:

(1.1) to evaluate the association between 18F-FDG PET/CT baseline semi-quantitative parameters of the primary lesion in advanced NSCLC patients eligible for immunotherapy with patient’s outcome (PFS and OS) and response to immunotherapy,

(1.2) to evaluate the application of radiomics analysis of the primary lesion for texture analysis to extract characteristics (quantitative biomarkers) from PET/CT images to represent tumor pathology or heterogeneity (phenotype),

(1.3) to evaluate if tumor burden assessed by 18F-FDG PET/CT, as nodal (N) and metastatic (M) status, is associated with PFS and OS.

### 2.2. Study Design and Inclusion Criteria

This is a retrospective, observational study approved by the ethical committee A.O.U. Città della Salute e della Scienza di Torino (IRB number: 0011604; code: IMMUNO PET-19). We analyzed the records and clinical/pathological characteristics of patients referred to Pulmonary Oncology Department, San Luigi Gonzaga Hospital, University of Turin. Eligible patients matched all the following inclusion criteria: (a) biopsy proven advanced NSCLC (stage IIIb/c or stage IV); (b) candidate to immunotherapy targeting the PD-1 or PD-L1; (c) 18F-FDG PET/CT performed at baseline, during the diagnostic work-up, within the start of immunotherapy. Patients with missing data and lost follow-up were excluded from the analysis. Compliance with ethical standards: Ethical approval for all procedures performed in studies involving human participants was in accordance with the ethical standards of the institutional and/or national research committee and with the principles of the 1964 Declaration of Helsinki and its later amendments or comparable ethical standards. Informed consent and local ethical committee approval: informed consent form (ICF) was obtained from all individual participants enrolled in this retrospective analysis. ICF has been provided.

### 2.3. ^18^F-FDG PET/CT Image Acquisition and Interpretation

Here, 18F-FDG PET/CT were performed with a standard protocol. Patients were instructed to fast for at least 6 h before the scan, and blood glucose levels were measured before the injection of 18F-FDG. Patients were excluded if their blood glucose levels at the time of the scans exceeded 150 mg/dL. Patient protocol included the intravenous injection of 2.5–3 MBq/kg of 18F-FDG, up to a maximum of 370 MBq (10 mCi), followed by a 60-min uptake period. All patients underwent PET/CT scan in a dedicated tomograph: AOU Città della Salute e della Scienza and IRCSS Istituto di Candiolo (Philips Gemini Dual-slice EXP, Philips Medical Systems, Cleveland, OH, USA)); Affidea-IRMET (Discovery 610, GE Healthcare, Chicago, IL, USA). An attenuation-corrected whole-body scan (base of the skull to mid thighs), 2 to 2.5 min per bed position (with an axial field-of-view of 18 cm per bed position), starting 60 min after tracer injection, was acquired. All patients performed a low-dose CT for attenuation correction and anatomical correlation of PET findings. The tomographs results were validated for a proper quantification and quality of the images recorded. The PET scans were reconstructed by ordered subset expectation maximization (OSEM)-based algorithms. All PET/CT images were analyzed with dedicated workstation (Advantage; GE Healthcare) and were independently interpreted with central reading by two nuclear medicine physicians, aware of clinical data.

### 2.4. PET Semi-Quantitative Parameters and Radiomics Analysis

Semi-quantitative PET parameters and radiomics features of the primary lesion were extracted using the open-access LIFEx platform v5.1 (IMIV/CEA, Orsay, France) [10]. For each primary lesion were evaluated PET semi-quantitative parameters, including maximum standardized uptake value (SUVmax), metabolic tumor volume (MTV) and total lesion glycolysis (TLG). The delineation of each primary lung lesion was performed manually, slice-by-slice, independently by two expert operators. Region of interest (ROI) with a threshold at 40% of SUVmax was also applied. MTV represents the volume of the above given ROIs. 3D ROIs based on percentage of SUVmax were applied (3D isocontour at 41% of the maximum pixel value [VOI41]). TLG was calculated as the product of the ROI average SUVmean multiplied by the corresponding MTV. In the longitudinal setting, the quantitative metrics described above were derived using the same approach for all PET/CT examinations in the same patient. Radiomics features were extracted from each of the two ROIs. The following radiomics features were analyzed: size and shape based–features such as volume, maximum diameter along different orthogonal directions, maximum surface, tumor compactness, and sphericity; first-order statistics features reporting the mean, median, maximum, minimum values of the voxel intensities on the image, as well as their skewness (asymmetry), kurtosis (flatness), uniformity, and randomness (entropy) and second-order statistics features include the so-called textural features. Lymph node and systemic metastasis were evaluated and grouped as N and M status.

### 2.5. Evaluation of the Response to Immunotherapy

Therapy response was routinely assessed by CT evaluation every 4 cycles of therapy and by clinical/laboratory evaluation. The diagnostic assessment of response to immunotherapy was performed with response evaluation criteria in solid tumors (RECIST) 1.1 criteria. Complete response (CR) was the disappearance of the primary tumor, partial response (PR) was a decrease of 30% or more in the longest diameter of the primary tumor, progressive disease (PD) was an increase of 20% or more in the longest diameter of the primary tumor, and stable disease (SD) was noted in patients whose tumors did not show either sufficient shrinkage to qualify for PR or a sufficient increase to qualify for PD [11,12]. When CT evaluation was not feasible for fast disease progression or the worsening of patient clinical condition, the response to immunotherapy was performed by clinical and laboratory evaluation only.

### 2.6. Outcome Measurements and Statistical Analysis

Demographic and baseline disease characteristics were recorded, censoring the times of observation on July 2019, at a median follow-up of 10 months (IQR 6–14 months). The primary endpoint was PFS, while the secondary one was OS. PFS was defined as the time from the start of immunotherapy to progression/death for any cause. OS was defined as the time from the start of immunotherapy to death as a result of any cause; patients still alive were censored at the date of last contact. The response to immunotherapy was assessed considering both clinical follow-up and RECIST 1.1 criteria.

Patient characteristics were compared using the Fisher’s exact test for categorical variables and the Mann–Whitney test for continuous ones. PFS and OS curves were estimated by the Kaplan–Meier method, comparing the two arms by the log-rank test and calculating 95% CIs. All p-values were obtained by 2-sided exact method at the conventional 5% significance level. All statistical analyses were performed in R 3.6.1.

Considering the radiomics analysis, univariate analysis with Kruskal–Wallis test was performed for each variable (histology, PDL-1 expression, disease stage, progression and response to therapy). The intraclass correlation coefficient (ICC) was used to assess intra- (by observer 1) and inter-observer (by observers 1 and 2) differences in texture and evaluate the reproducibility of textural parameters. The ICC was also used to assess differences in texture between manual and semi-automatic segmentation.

## 3. Results

### 3.1. Population Characteristics

Fifty-seven patients have been retrospectively enrolled: 17 females and 40 males, with a median age of 69 years old (range 39–84 years old). Three out of 57 patients were non-smokers, 33/57 were former smokers, while 21/57 were current smokers. Sixty-six percent (38/57) of patients had adenocarcinoma (AC), 17.5% (10/57) squamous cell carcinoma (SCC) and 15.7% (9/57) were not otherwise specified (NOS). According to the 8th Edition of TNM in Lung Cancer (8), about half of the patients (47.4%) were stage IVa, 42.1% were IVb and 8.8% of patients were IIIb. The Eastern Cooperative Oncology Group, performance status (ECOG PS) was 0 in 20/57 patients, 1 in 35/57 and two patients had an ECOG PS of 2. All patients were wildtype (no EGFR mutations, no ALK rearrangements detected). The PD-L1 expression on the bioptic specimen was evaluated in 51/57 patients. Notably, 80.4% of patients (41/51) presented a PD-L1 expression higher than 50%, 7.8% (4/51) between 1 and 49% and 11.8% (6/51) patients lower than 1%. All patients’ characteristics are listed in Table 1.

### 3.2. Immunotherapy Scheme

Treatment was decided by referent physicians considering tumor histology, PD-L1 expression and patients’ risk factors. Immunotherapy was performed as a frontline therapy in 42/57 (73.7%) patients and as a second line therapy after chemotherapy in 15/57 (26.3%) patients. The formers were all treated with Pembrolizumab after the assessment of PD-L1 expression higher than 50%. The latter were treated with Pembrolizumab in 5 cases, Nivolumab in 5 cases and Atezolizumab in 5 cases as well. Of these 15 cases treated with immunotherapy as second line, 8 patients received platinum + pemetrexed, 5 patients platinum + gemcitabine and 2 patients platinum + docetaxel. Six out of fifteen patients underwent surgery on the primary lesion before starting immunotherapy. Pembrolizumab, nivolumab and atezolizumab were administered intravenously at the standard dose.

### 3.3. Follow-Up and Therapy Response to Immunotherapy

The median follow-up was 10 months (range 1.5–68.6; IQR 6–14 months). Forty percent (23/57) of patients died from cancer-related causes, while 59.6% (34/57) were alive with evidence of disease. At the last follow-up, 30 patients were PD, 19 were SD and 8 were PR. No CR was observed. Thirty patients (52.6%) experienced disease progression during therapy. Among patients with progressive disease, 21 had the appearance of new lesions on CT performed as part of monitoring therapy response (RECIST 1.1). In 9 cases, CT evaluation was not feasible (fast disease progression or worsening of patient clinical condition) and disease progression was observed by clinical and laboratory evaluation.

Four patients experienced immunotherapy-induced toxicity (skin reaction, cough and rising liver enzymes) and four patients experienced acute events (transient ischemic attack, pneumonia and acute respiratory failure).

Immunotherapy was suspended in 30/57 patients. The causes of suspension were: progression (clinical and/or radiological) of disease in 27 cases assessed through CT and/or clinical evaluation, severe toxicity in 2 cases and death in 1 case. In 25/57 patients, immunotherapy was ongoing at the time of the last follow-up. The median number of cycles of therapy was 7 (mean = 11; range 1–69).

### 3.4. ^18^F-FDG PET/CT Findings

First, 18F-FDG PET/CT was performed as routine diagnostic procedure to stage the disease before the start of immunotherapy. The median time from PET/CT and the start of immunotherapy was 37 days (mean = 45; range 4–133 days). All primary tumors, observed in the 51/57 patients who did not receive surgery and/or radiation therapy with radical intent, exhibited increased FDG uptake.

Twenty-two patients were N0, while 35/57 had positive lymph nodes (N+). In details, 10 were N1 (ipsilateral peribronchial and/or hilar and intrapulmonary), 12 patients were N2 (ipsilateral mediastinal and/or subcarinal nodes), and 13 patients were N3 (contralateral mediastinal or hilar nodes or supraclavicular nodes). Systemic metastases were seen in 34/57 patients, while 23 patients were staged as M0. 18F-FDG PET/CT observed adrenal metastases in 10 cases, contralateral lung nodules in 9, bone metastases in 9, liver lesions in 6, pleural involvement or malignant effusion in 5, extra-pulmonary lymph nodes in 2, soft tissue localization in 2 cases and 1 mesenterial nodule. Eleven patients were classified as M1a (contralateral lung or pleural/pericardial nodule or effusion), six were M1b (single extra-thoracic metastasis) and seventeen were M1c (multiple extra-thoracic metastases).

### 3.5. PET Semi-Quantitative Parameters Analysis (Objective 1.1)

**Figure 1:**
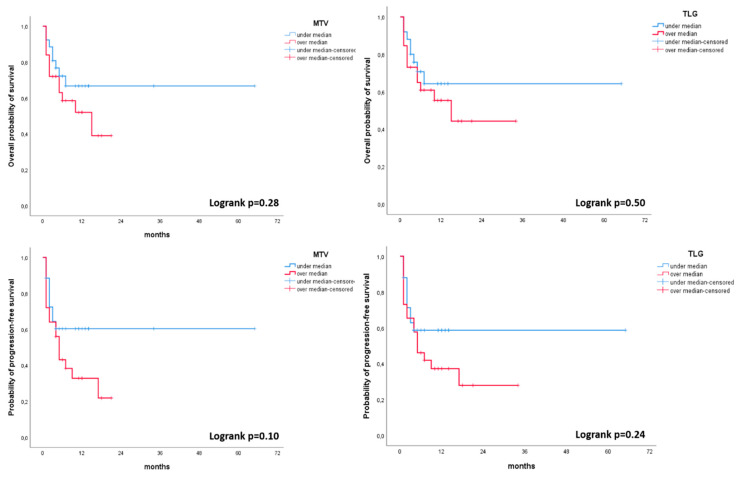
Kaplan–Meier plot analysis for MTV and TLG with both progression free survival (PFS) and overall survival (OS). Population was grouped by the median value of MTV (74.1) and TLG (310.8).

The values of baseline 18F-FDG PET/CT semi-quantitative parameters of the primary lesion are listed in Table 2. At the Mann–Whitney test, MTV (*p* = 0.028) and TLG (*p* = 0.035) were significantly associated with progressive vs. non-progressive disease status (Table 3). Patients with higher values of MTV and TLG of the primary lesion at the baseline PET/CT had higher probability of disease progression, compared to those patients presenting with lower values. SUVmax values did not show significant association between the two groups (PD vs. non-PD patients) (*p* = 0.55). In the Kaplan–Meier plot analysis, the variables resulted associated with PD vs. non-PD status (MTV and TLG) were compared with PFS and OS (Figure 1). MTV and TLG were stratified according to the median value: upper and lower median value (MTV = 74.1 ml3; TLG = 310.8). Kaplan–Meier curves showed a trend to predict disease progression among patients with MTV and TLG lower or higher the median values. However, the log rank test of the two curves showed them not to be significantly different (Figure 1). In patients with MTV and TLG upper median values, PFS was 5 months, while OS was 15 months. In patients with MTV and TLG lower median values, PFS and OS were not reached (minimum 50% of events at last follow-up not reached).

In the Kruskal–Wallis test, MTV (*p* = 0.027) and TLG (*p* = 0.022) resulted in being significantly different among PR, SD and PD groups (Table 4). SUVmax was confirmed to not be associated with response to therapy criteria (*p* = 0.427) (Table 4).

### 3.6. Radiomics Analysis (Objective 1.2)

The intraclass correlation coefficient (ICC) analysis of the texture parameters of the primary lesion, based on two different delineations generated by two observers, showed that all parameters presented good reproducibility (ICC > 0.95), except for GLZLM_SZLGE (ICC = 0.37). However, when applying a semi-automatic segmentation (threshold = 40% of the SUVmax), only 8 features showed good reproducibility (SUVmax, sphericity, two first-order statistics features and 4 textural features. ICC > 0.95). The univariate analysis with the Kruskal–Wallis test was performed for the following variables: histology, PDL-1 expression, disease stage, progression and response to therapy.

**Figure 2:**
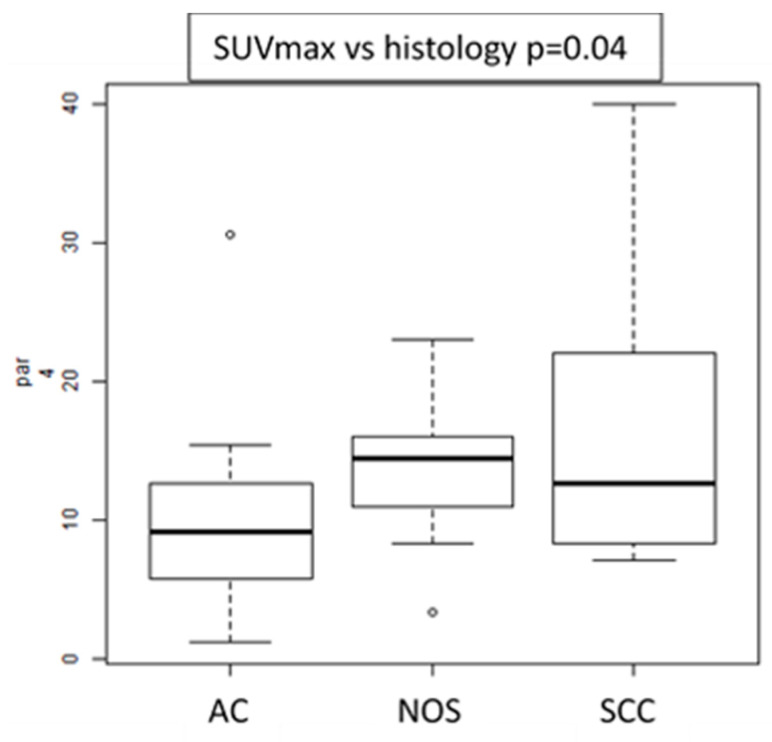
Box plot of SUVmax values showing differences between histological subtypes.

Histological variants: the only feature associated with different histological subtypes was SUVmax (Figure 2). Namely, SCC showed higher SUVmax values than AC and NOS subtype.

**Figure 3:**
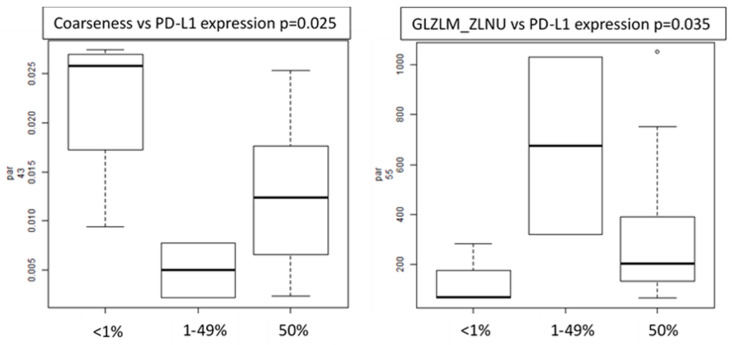
Box plots of radiomics features (coarseness and GLZLM_ZLNU) showing correlation with PD-L1 expression.

PD-L1 expression: several features (including SUVmax, volume, busyness, coarseness, GLZML_ZLNU and GLZML_GLNU) resulted in being significantly different between patients with PD-L1 expression lower than 1%, between 1% and 49% and higher than 50%. Considering the semi-automatic ROIs, only two features (coarseness and GLZLM_ZLNU) maintained the statistical correlation (Figure 3).

Disease stage: radiomics features did not show significant difference between IIIb, IVa and IVb patients at both manual and semi-automatic segmentation.

**Figure 4:**
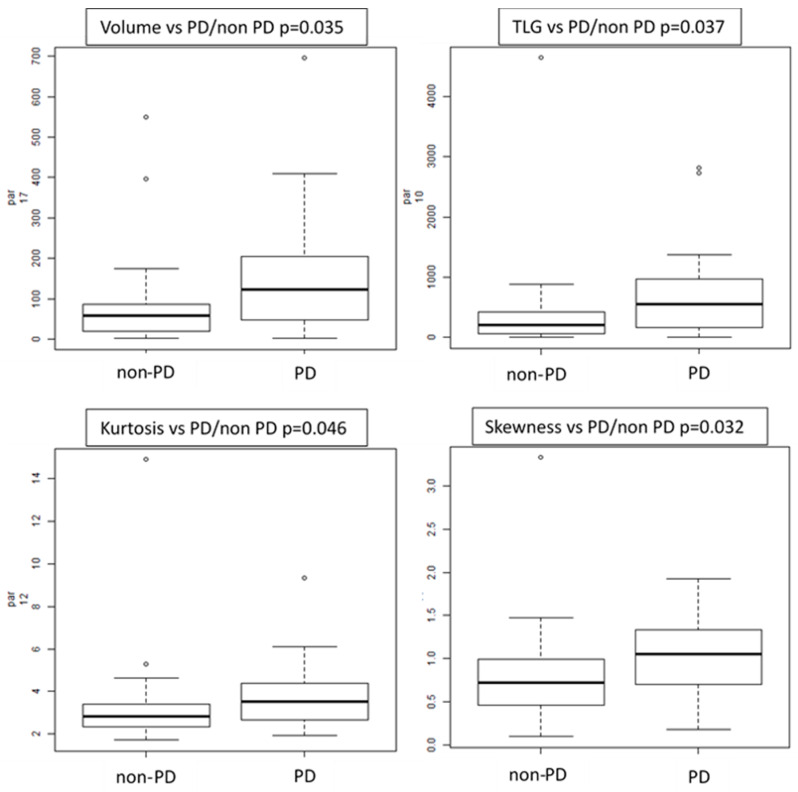
Box plots of radiomics features correlated with progressive (PD) vs. non-progressive disease status (non-PD).

Progression status: several features showed differences between PD vs. non-PD patients, such as volume (*p* = 0.035), TLG (*p* = 0.037), three first-order histograms-based features (kurtosis, excess-kurtosis and skewness) and six textural features (GLZLM_LZE, GLRLM_RP, GLRLM_SRE, GLRLM_HGRE, GLRLM_SRHGE and GLCM Homogeneity) (Figure 4). GLZLM represents the grey-level zone length matrix and provides information on the size of homogeneous zones for each grey-level in 3 dimensions. GLZLM_LZE is the distribution of long homogeneous zones in an image. GLRLM represents the size of homogeneous runs for each grey level. “Run” means the presence of elements in the image with the same gray-level on in a row/line. GLCM_homogeneity considers the arrangements of pairs of voxels to calculate textural indices and represents the homogeneity of grey-level voxel pairs.

Accordingly, patients with primary lung tumor presenting with higher TLG value, higher volume, higher asymmetry and kurtosis had higher probability to progress.

Response to therapy: TLG, volume and two textural features showed a significant difference between PR, SD and PD patients at both manual and semiautomatic evaluation.

### 3.7. Tumor Burden Analysis (Objective 1.3)

**Figure 5:**
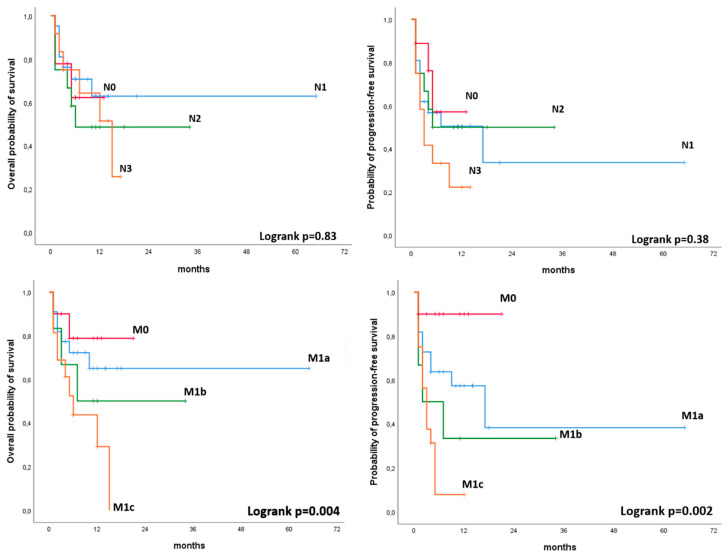
Kaplan–Meier plot analysis for N status (N0, N1, N2, N3) and M status (M0, M1a, M1b, M1c) with PFS and OS.

In the Kaplan–Meier plot analysis, M status (M0, M1a, M1b, M1c) as assessed by 18F-FDG PET/CT was significantly associated with PFS (*p* = 0.002) and OS (*p* = 0.049). Namely, patients with no visible metastases at PET/CT had significantly better outcome compared with those patients presenting with FDG avid metastases. No significant associations were observed for N status (N0, N1, N2, N3) as assessed by 18F-FDG PET/CT and PFS or OS. (Figure 5).

## 4. Discussion

Over the past decade 18F-FDG PET/CT demonstrated to be a powerful tool for staging and assessing treatment response in patients suffering from NSCLC [13,14]. Even if PET/CT is widely used in this clinical scenario, limited data are currently available regarding the role of PET/CT semi-quantitative parameters as predictors of patient outcome [15,16]. Findings are generally heterogeneous and this variability might be probably related to the heterogeneity of the population enrolled. Furthermore, the association of semi-quantitative parameters with patients’ outcome can be related to the clinical stage of the disease (locally vs. advanced disease) and to the therapy investigated (chemo-radiotherapy vs. biological therapy) [15,16,17,18]. At present, SUV values, MTV and TLG are the PET-derived parameters used to assess the tumor metabolic activity in NSCLC [18]. MTV and TLG can easily be calculated in the primary tumor by means of a segmentation technique. The manual or semi-automatic measurement of the pre-treatment MTV has been shown to be better than SUVmax for predicting patients’ prognosis in different solid neoplasms such as head and neck cancer, with or without metastases [19,20].

Our study aimed to investigate the relationship between the functional tumor parameters at baseline (SUVmax, MTV, and TLG) and PFS and OS in patients with advanced NSCLC, presenting with stage IIIB, IIIC, IVa and IVb, for whom immunotherapy was shortly planned. A significant association of MTV and TLG of the primary lesions with PD was observed, since lower MTV and TLG values were associated with non-PD status; while, on the contrary, primary lesions presenting with higher values were more likely associated with the progression of the disease during immunotherapy. On the contrary, SUVmax did not show significant association with both PFS and OS. These results are in accordance with recent studies, suggesting a superior correlation of volumetric 18F-FDG PET/CT parameters, rather than SUVmax only [7,9,21,22]. However, despite the fact that in the Kaplan–Meier analysis, MTV and TLG showed a trend in predicting disease progression, the curves associated with PFS and OS were not statistically significant. This is probably due to the relatively small sample size and the limited number of events that occurred.

Grizzi et al., in a preliminary analysis on 27 patients with NSCLC, found an antithetical correlation between 18F-FDG PET/CT pre-treatment parameters and the response to immunotherapy. Authors observed a rapid progression of disease after 8 weeks from the start of immunotherapy in patients with a SUVmax up to 17.1 and a SUVmean up to 8.3, with a sensitivity of 88.9% and 100%, respectively [23]. Similarly, Evangelista et al. investigated the role of semi-quantitative 18F-FDG PET/CT parameters in NSCLC treated with Nivolumab. Authors observed an association between the sum of metabolic parameters of all lesions and PD status, with SUVmax having the strongest association [24]. Their data seem to be in contrast with our analysis. However, authors considered all the lesions together as the cumulative sum of all FDG deposits detected by PET/CT, while we specifically designed our study to analyze semi-quantitative parameters of the primary lesion only, and we analyzed the correlation of the tumor burden (N and M status assessed by 18F-FDG PET/CT) separately, with a dedicated objective (objective 1.3). In our study, MTV and TLG were also significantly associated with immunotherapy response. Patients with SD or PR had lower values of MTV and TLG compared to patients with PD. No correlation was observed again between SUVmax values and response to therapy. Accordingly, since MTV and TLG are parameters that accurately reflect the glycolytic metabolic status of a lesion and thus the disease aggressiveness, 18F-FDG PET/CT might be considered as a prognostic tool to stratify patients presenting with higher metabolic primary tumors that, most likely, will fail immunotherapy. In our study, we also observed a significant association between the M status at 18F-FDG PET/CT and PFS and OS. It is worth noting that 18F-FDG PET/CT offers the possibility of having a single-step whole body examination that can clearly identify distant metastases and select NSCLC patients treated with immunotherapy with a higher probability of experiencing rapid disease progression and poor survival outcomes.

Immunotherapy has completely changed the prognosis and the care of patients with NSCLC. However, the benefit with immunotherapy is not seen for the entire population, making the identification of biomarkers a critical step in selecting the candidate population. Therefore, radiomics aims to identify biomarkers that better predict patient response [25,26]. Radiomics has been investigated in various studies to identify clinical and computational image-based predictors of rapid disease progression phenotypes in NSCLC patients [7,27,28]. However, there are only few studies investigating the application of this new field in patients with NSCLC treated with checkpoint-based immunotherapy and most of them based radiomics analysis on CT images and enrolled heterogeneous population with different primaries (e.g., lung and melanoma) [29,30]. Recently, Mu et al. presented promising data and a novel methodological approach on the application of radiomics analysis in NSCLC treated with immune checkpoint blockades. In their study, authors tested the hypothesis that radiomics features from baseline pretreatment 18F-FDG PET/CT scans can predict the clinical outcomes of NSCLC patients treated with checkpoint blockade immunotherapy. Among other findings, authors reported that when investigating the informative components of multi-parametric radiomics signature (mp-RS) formula, they found that multiple texture features (PET_SRLGE, KLD_SZE) positively correlated, suggesting that the more heterogeneous tumors had a larger probability to have a durable clinical benefit (DCB). These results are slightly in contrast with prior studies, which showed that more heterogeneous tumors with CT textures had worse response to radiation or chemotherapy [31]. Our results were slightly in contrast with Mu et al. [7] as well. In our study, the most interesting difference of features was seen between patients with PD and patients with non-PD. Namely, patients with primary lung tumor presenting with high TLG value, high volume and tumor heterogeneity represented by asymmetry (skewness feature) and kurtosis (how heavily the tails of a distribution differ from the tails of a normal distribution) had a higher probability of progression and were associated with non-response to immunotherapy. However, differences in features extraction methodology and different endpoints (DCB vs. PD) make these findings not completely comparable. Finally, segmentation is currently the rate-limiting step in radiomics analysis. To overcome this issue, in our study, the segmentation of the ROIs was performed manually by two independent operators and semi-automatically, applying a threshold of 40% of the SUVmax.

### Limitation

This work is not exempt from limitations. The retrospective design of this analysis might have probably affected population selection. A prospective design would probably offer the chance to recruit a more homogenous population (e.g., same immunotherapy scheme, shorter range for PET scan before immunotherapy, immunotherapy administrated as first-line). Namely, in our cohort, 26.3% of patients received immunotherapy after platinum-based chemotherapy. However, the population enrolled represents the typical cohort of patients generally referred to 18F-FDG PET/CT before immunotherapy in daily clinical practice.

This analysis was performed in a relatively small sample size. A larger cohort would be preferable. In our study, we observed an association of MTV and TLG with PD vs. non-PD status. Nevertheless, this association was not maintained in the Kaplan–Meier analysis and, despite a non-negligible trend, MTV and TLG were not statistically significantly associated with PFS. The limited number of events at the end of follow-up, namely for patients with lower than median values for MTV and TLG, influenced the correlation between these metabolic parameters and PFS and OS. It is probable that our statistical model might rich significance in a larger cohort.

Another limitation is related to the lack of “early intermediate” PET performed while immunotherapy was still on-going, in order to evaluate PET response to therapy. However, since the role of PET imaging during immunotherapy is not yet established, 18F-FDG PET/CT is generally performed in clinical practice at baseline only. Thus, none of the patients enrolled in our study performed a second PET scan.

Considering radiomics analysis, the ideal setting would require a robust validation regarding reconstruction and segmentation on different tomographs. In our analysis, despite similar 18F-FDG PET/CT protocols and using the same reconstruction algorithm, the texture analysis was performed on images derived by three different tomographs. A common issue with radiomics analysis is represented by segmentation process (manual vs. automatic) and a comparison between the two approaches would be preferable. However, to strengthen our analysis, we included two operators who independently perform the segmentation. Furthermore, a threshold of 40% of SUVmax was also applied to both operators’ ROI. Finally, in our study, segmentation was performed on PET images only, while low-dose CT images were not segmented, and so not considered in our radiomics analysis.

## 5. Conclusions

According to our results, baseline 18F-FDG PET/CT performed before the start of immunotherapy might be important, since it offers the chance to evaluate different quantitative parameters potentially associated with different response to therapy in patients with advanced NSCLC. In our study, and considering the limitations exposed above, MTV, TLG and radiomics features (volume and heterogeneity) were associated with disease progression, while metastatic tumor stages assessed with PET/CT were associated with different patients’ outcome.

These results, while far from definitive evidence, encouraged us to initiate a prospective trial to investigate the role of radiomics features extracted from 18F-FDG PET/CT in NSCLC eligible to immunotherapy.

## Figures and Tables

**Table 1:** Overall population (*n* = 57) characteristics.

Characteristics	Value
**Age**	Median 69 years old (range 39–84)
**Sex**	
Male	40/57 (70.2%)
Female	17/57 (29.8%)
**Smoking Status**	
Non-smoker	3/57 (5.3%)
Ex-smoker	33/57 (57.9%)
Current smoker	21/57 (36.8%)
**Histological Variant**	
Adenocarcinoma	38/57 (66.6%)
Squamous cell carcinoma	10/57 (17.5%)
NOS	9/57 (15.7%)
**ECOG PS**	
0	20/57 (35.1%)
1	35/57 (61.4%)
2	2/57 (3.5%)
**Stage at Diagnosis**	
IB	1/57 (1.75%)
IIB	1/57 (1.75%)
IIIA	5/57 (8.8%)
IIIB	7/57 (12.3%)
IIIC	1/57 (1.7%)
IVA	26/57 (45.6%)
IVB	16/57 (28.1%)
**Stage before IO**	
IIIB	6/57 (8.8%)
IVA	27/57 (47.4%)
IVB	24/57 (42.1%)
**PD-L1 expression analysis**	
Not Performed	6/57 (10.5%)
<1%	6/57 (10.5%)
1–49%	4/57 (7.0%)
>50%	41/57 (72.0%)
**Immunotherapy**	
First line IO	42/57 (73.7%)
Second line IO	15/57 (26.3%)

NOS: Not Otherwise Specified; ECOG PS: Eastern Cooperative Oncology Group Performance Status; IO: Immuno-Oncology therapy.

**Table 2:** In this table; 18F-FDG PET/CT semi-quantitative parameters: SUVmax, metabolic tumor volume (MTV) and total lesion glycolysis (TLG) values.

PET Parameters	Minimum	Percentile 25	Median	Percentile 75	Maximum
SUVmax (g/mL)	1.4	5.8	9.5	13.5	30.5
MTV (mL^3^)	3.1	36.7	74.1	163.3	695.2
TLG	2.8	87.3	310.8	852.9	4639.8

**Table 3:** SUVmax, MTV and TLG values among patients with progressive (PD) and non-progressive (non-PD) disease.

Associations Between Baseline PET Parameters and PD vs. non-PD							
PET Parameters	Status	Minimum	Percentile 25	Median	Percentile 75	Maximum	*p* Value
SUVmax (g/mL)	PD	2.5	5.8	11.5	13.6	30.5	0.55
	non-PD	1.4	5.9	9.3	13.4	28.9	
MTV (mL^3^)	PD	3.1	47.0	124.4	203.1	695.2	0.028
	non-PD	3.1	19.7	57.4	86.6	548.5	
TLG	PD	4.5	165.9	536.1	988.1	2820.3	0.035
	non-PD	2.8	55.0	205.8	417.8	4639.8	

**Table 4:** Association of MTV and TLG with response to immunotherapy, as assessed by response evaluation criteria in solid tumors (RECIST) 1.1 criteria and clinical follow-up.

PET Parameters	Associations Between Baseline PET Parameters and Response to Immunotherapy						
	Status	Minimum	Percentile 25	Median	Percentile 75	Maximum	*p* Value
SUVmax (g/mL)	PR	1.4	5.8	8.8	13.2	15.3	0.427
	SD	5.7	9.2	11.9	15.0	28.9	
	PD	2.5	6.4	10.7	13.5	30.5	
MTV (mL^3^)	PR	3.1	15.3	49.5	79.6	173.9	0.027
	SD	26.9	72.2	75.0	375.9	548.5	
	PD	3.1	44.3	124.3	202.6	695.2	
TLG	PR	2.8	30.4	139.3	251.3	865.6	0.022
	SD	83.4	310.8	417.8	852.9	4639.8	
	PD	4.5	151.3	440.8	979.9	2820.3	
